# Isolation and characterization of nutrient dependent pyocyanin from *Pseudomonas aeruginosa* and its dye and agrochemical properties

**DOI:** 10.1038/s41598-020-58335-6

**Published:** 2020-01-31

**Authors:** Savitha DeBritto, Tanzeembanu D. Gajbar, Praveen Satapute, Lalitha Sundaram, Ramachandra Yarappa Lakshmikantha, Sudisha Jogaiah, Shin-ichi Ito

**Affiliations:** 1 Laboratory of Plant Healthcare and Diagnostics, P.G. Department of Biotechnology and Microbiology, Karnatak University, Dharwad, Karnataka 580003 India; 2 Division of Biological Sciences, School of Science and Technology, The University of Goroka, Goroka, 441 Papua New Guinea; 3 Department of Botany, Periyar Palkalai Nagar, Periyar University, Salem, 636011 Tamil Nadu India; 4 Department of Biotechnology and Bioinformatics, Kuvempu University, Jnanasahyadri, Shivamogga, 577451 India; 5 Laboratory of Molecular Plant Pathology, Department of Biological and Environmental Sciences, Graduate School of Sciences and Technology for Innovation, Yamaguchi University, Yamaguchi, 753-8515 Japan; 6 Research Center for Thermotolerant Microbial Resources (RCTMR), Yamaguchi University, Yamaguchi, 753-8515 Japan

**Keywords:** Biotechnology, Microbiology, Plant sciences

## Abstract

Pyocyanin is a blue green phenazine pigment produced in large quantities by active cultures of *Pseudomonas aeruginosa*, with advantageous applications in medicine, agriculture and for the environment. Hence, in the present study, a potent bacterium was isolated from agricultural soil and was identified morphologically and by 16S rRNA sequencing as *P*. *aeruginosa* (isolate KU_BIO2). When the influence of nutrient supplements in both King’s A and Nutrient media as amended was investigated, an enhanced pyocyanin production of 2.56 µg ml^−1^ was achieved in King’s A medium amended with soya bean followed by 1.702 µg ml^−1^ of pyocyanin from the nutrient medium amended with sweet potato. Purified pyocyanin was characterized by UV-Vis Spectrophotometer and Fourier-Transform Infrared spectroscopy (FTIR). Furthermore, Liquid Chromatography Mass Spectrum (LCMS) and Nuclear Magnetic Resonance (NMR) confirmed its mass value at 211 and as N-CH_3_ protons resonating at 3.363 ppm as a singlet respectively. The isolated pyocyanin displayed remarkable dye property by inducing color change in cotton cloth from white to pink. Lastly, the antifungal activity of test pyocyanin showed inhibition of growth of rice blast fungus, *Magnaporthe grisea* and bacterial blight of rice, *Xanthomonas oryzae* at concentrations of 150 and 200 ppm, respectively. Thus, this investigation provides evidence for diverse actions of pyocyanin which are nutrient dependent and are capable of acting on a large scale, by utilizing microbes existing in agriculture wastes, and thus could be used as an alternative source in the making of natural textile dyes with strong durability and a broad spectrum of ecofriendly agrochemicals.

## Introduction

Microorganisms are biological agents which help to solve many problems related to health, agriculture and the environment^1–3^. Microorganisms have been the study at interest in recent years because of production of novel secondary metabolites^4^. These metabolites exhibit antimicrobial, antiviral or antitumor as well as anticoagulant properties, and the production of secondary metabolites may have evolved as an alternative strategy to switching off metabolic pathways by various control mechanisms^5–7^. Products of secondary metabolism, such as pigments, could also be of considerable selective advantage since they could eliminate possible microbial competitors^8^.

Natural pigments have been obtained and used since long ago, but interest in them has decreased due to toxicity problems. Hence, pigments from microbial sources are good alternatives for various applications^9,10^. *Pseudomonas aeruginosa* is one of the most commercially valuable organisms, many of which are responsible for producing soluble pigments like pyocyanin (blue), pyoveridin (yellow-green), pyorubin (red) and pyomelanin (brown)^11^. *P*. *aeruginosa* produces pyocyanin (N- methyl- 1- hydroxyphenazine) which is a water soluble blue-green phenazine color pigment produced in large quantities^12^. Despite of its antimicrobial and other commercial significant properties, there are reports that the bacterium *P*. *aeruginosa* are prominent pathogen in nature that cause various health issues especially in hospitalized patients^13,14^.

Pyocyanin is a potent bacterial pigment produced by *P*. *aeruginosa* with capacity to arrest the electron transport chain of fungi and hence exhibits antifungal activity^15^. Additionally, pyocyanin is a broad spectrum pigment which acts on pathogenic microbes, importantly on wilt disease which is caused by *Fusarium oxysporum*^16^. In addition to its diverse applications in the agriculture field, pyocyanin is used also in textile industries as a colorant agent for cotton and linen cloths^17,18^. Therefore, the main purposes of this study are (i) to identify and isolate, the pyocyanin producing organism *P*.*aeruginosa* from agricultural waste soil (ii) to test productivity of pyocyanin under different nutrients and (iii) characterization of pyocyanin and its evaluation on both the dye and agrochemical properties.

## Results

### Isolation and characterization of pyocyanin producing bacteria

**Figure 1:**
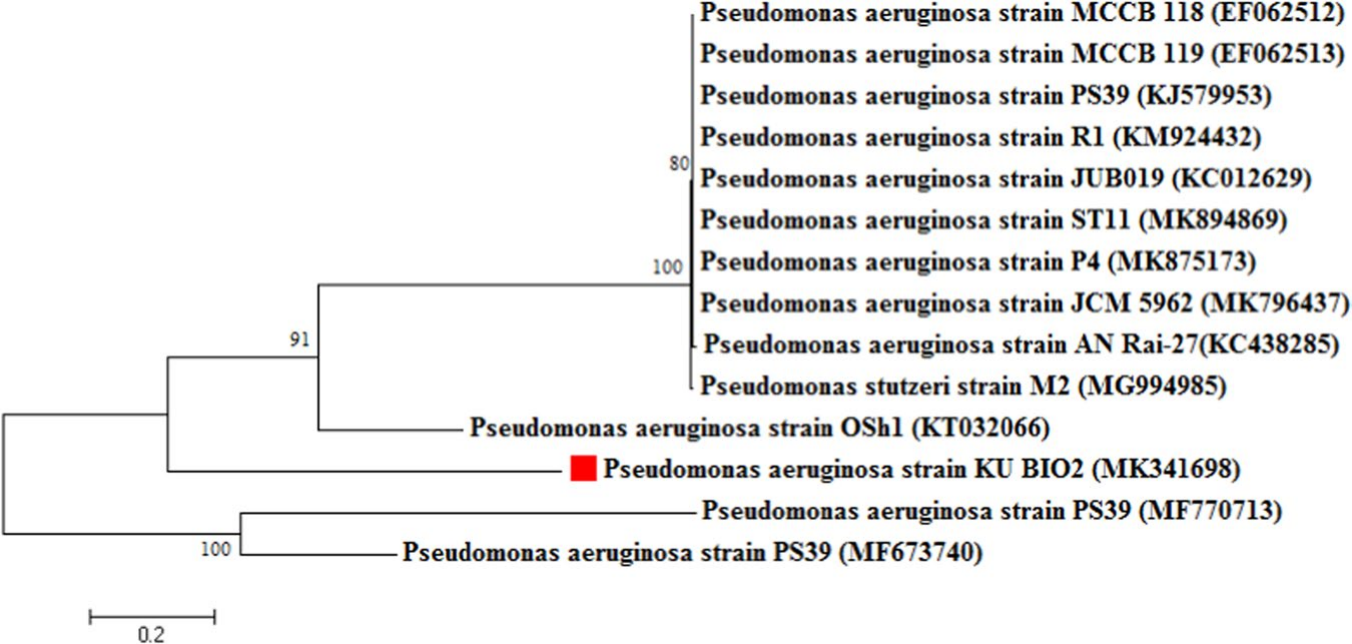
Phylogenetic tree of 16S rRNA gene showing resemblance to various isolates of pyocyanin producing bacterium *Pseudomonas aeruginosa* drawn using MEGA 7.0 software with neighbor joining method (NJ) cluster analysis (numbers inside the branches are bootstrap values).

A total of seven strains of bacteria designated as KU_BIO1-KU_BIO7 were isolated from the soil. Among the seven strains, KU_BIO2 produced a fluorescent glow and this strain was used for further study. The isolated strain (KU_BIO2) was found to be an aerobic Gram negative bacterium. The fully developed colonies of the strain KU_BIO2 were round in shape with raised elevation. Further, this strain showed positive results for catalase, oxidase and citrate test. The 16S rRNA sequence acquired was 971 bp long, and the isolate was identified as *Pseudomonas aeruginosa* KU_BIO2 strain and deposited in the National Center for Biotechnology Information (NCBI) GenBank under the accession number MK341698. The nearest relatives were first decided upon based on comparability of their 16S rRNA sequences obtained by a direct blast analysis of the NCBI GenBank. The phylogeny of strain KU_BIO2 was identified by retrieving the *Pseudomonas* spp. which were classified as able members in pyocyanin production. Based on the phylogeny, the strain KU_BIO2 exhibited 100% similarity with MK875173 and MK796437 which are potent bacteria for rhizodegradation of used engine oil and biosurfactants production respectively. Overall, the genetic sequence of the isolated strain (KU_BIO2), when blast searched in NCBI, showed high similarity with non-pathogenic bacteria, further none of the pathogenic isolates of *P*. *aeruginosa* found in the NCBI database showed similarity with this isolate (KU_BIO2) (Fig. 1).

### Morphological analysis of the strain by Atomic Force Microscopy (AFM)

**Figure 2:**
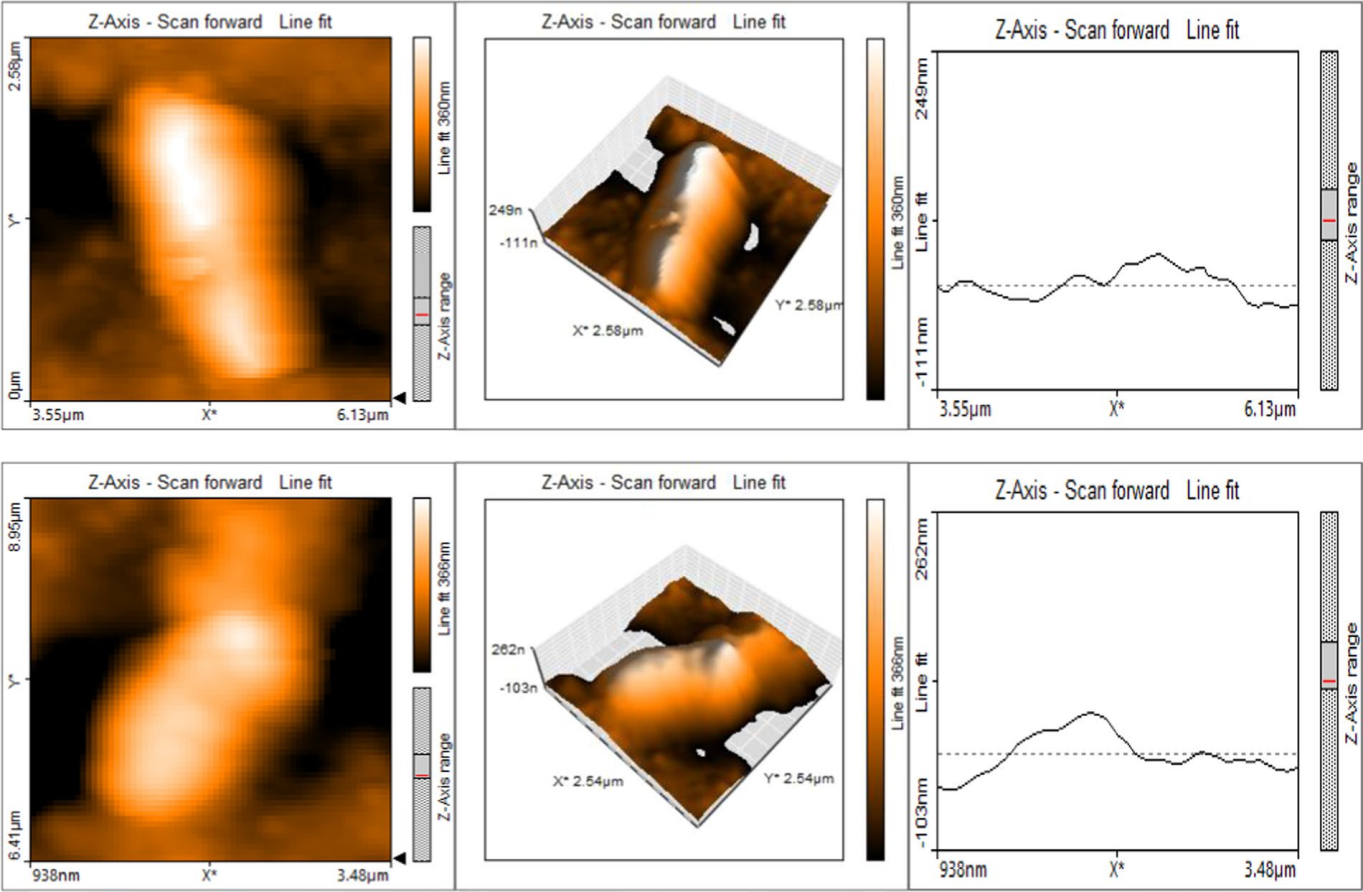
Cell morphology of *Pseudomonas aeruginosa* strain KU-BIO2 displaying the surface area as analyzed by Atomspheric Fluoresent Microscopy captured at various angles.

The cells of *P*. *aeruginosa* KU-BIO2 strain were scanned between 0 to 2.5 µm and it was observed that the single cell was found to be approximately 1.9 µm in length and 0.26 µm of width as illustrated in Fig. 2.

### Production of pyocyanin on different broths

The King’s A broth amended with soya as a nutrient yielded maximum pyocyanin followed by the nutrient broth amended by sweet potato. The maximum production in nutrient broth was observed in the sweet potato broth with a concentration 1.702 µg ml^−1^ and the minimum production was observed in the soya bean broth with a concentration 0.1702 µg ml^−1^. The details of pyocyanin production are tabulated in Table 1. The maximum production in King’s A broth was observed in the soya bean broth with a concentration 2.560 µg ml^−1^. The minimum production was observed in the watermelon broth with a concentration 0.5106 µg ml^−1^ (Table 2).

**Table 1:** Production of pyocyanin in nutrient broth amended with different nutrition sources.

Sample name	Optical density at 520 nm	Concentration (µg ml^−1^)
Corn	0.02	0.3414 ± 0.012^d^
Soya bean	0.01	0.1702 ± 0.001^de^
Sweet potato	0.10	1.702 ± 0.031^a^
Watermelon seeds	0.08	1.3616 ± 0.0011^b^
Groundnut	0.05	0.851 ± 0.003^c^

**Table 2:** Production of pyocyanin in King’s A broth amended with different nutrition sources.

Sample name	Optical density at 520 nm	Concentration (µg ml^−1^)
Corn	0.11	1.877 ± 0.034^b^
Soya bean	0.15	2.560 ± 0.021^a^
Sweet potato	0.10	1.702 ± 0.018^b^
Watermelon seeds	0.03	0.5106 ± 0.015^cd^
Groundnut	0.05	0.851 ± 0.009^c^

### Chemical analysis

#### Confirmative test for the production of pyocyanin using UV spectroscopy

**Figure 3:**
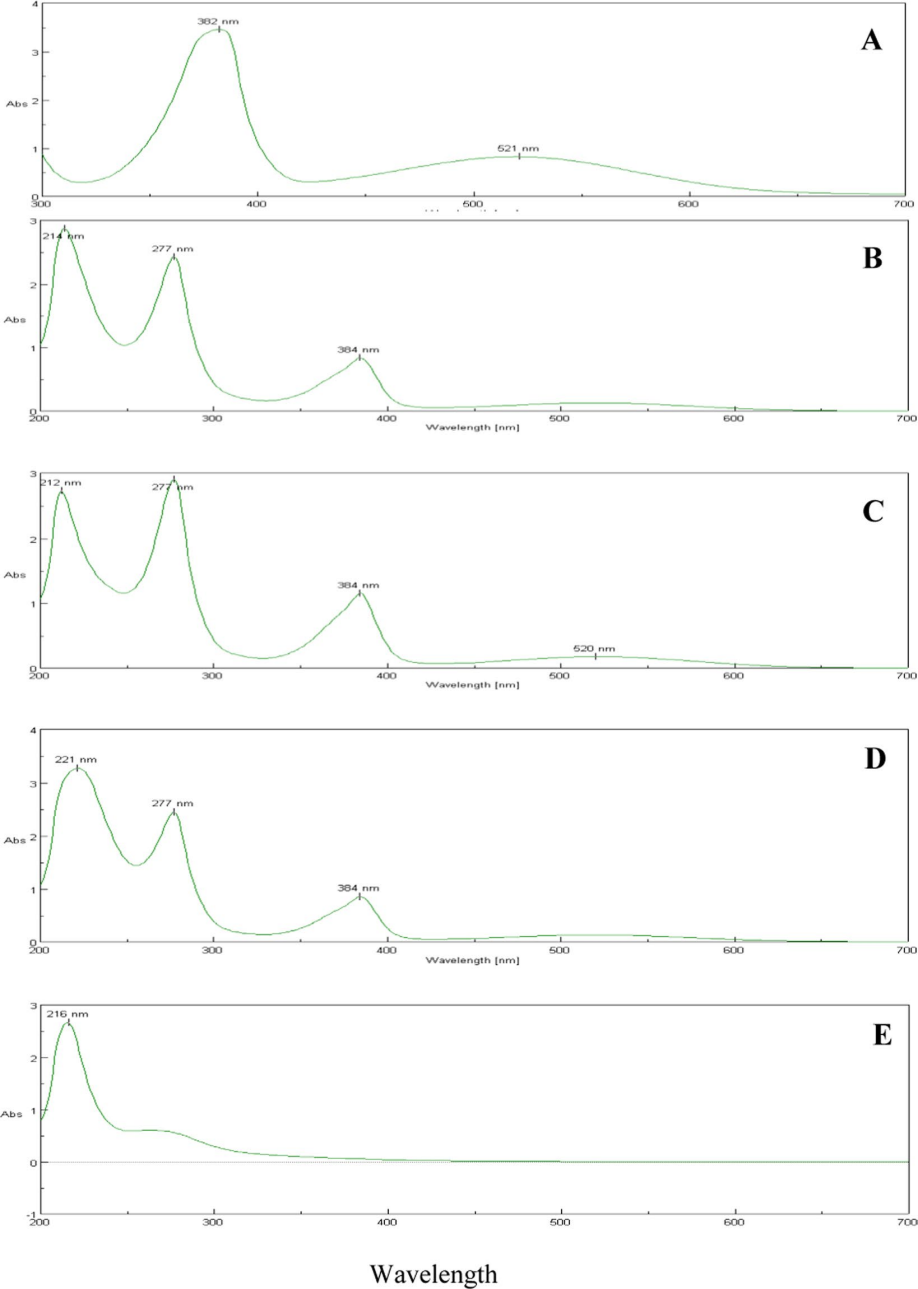
Spectroscopic absorbance peaks of standard pyocyanin (**A**) and pyocyanin extracted from the King’s A medium amended with corn (**B**), soya (**C**), sweet potato (**D**) and water melon (**E**).

The absorbance spectra of pyocyanin were measured from 200 to 700 nm wavelength. The absorbance peaks of standard pyocyanin were eluted at 382 and 521 nm against 0.2 M HCL as a blank (Fig. 3). Similarly, the absorbance peaks eluted from King’s A broth extracts with five different supplements were 214, 277 and 384 nm for the corn and sweet potato, 212, 277, 384 and 520 nm for the soya bean and 216 nm for the watermelon seeds respectively (Fig. 3). Hence by comparing all the peaks in the King’s A broth, the soya bean has a higher absorbance peak than those of the other raw materials used. The maximum production range of pyocyanin in the King’s A broth was at 212, 277, 384 and 520 nm in the soya bean and the minimum production was at 214 nm in the groundnut broth. Similarly, the absorbance peaks seen in the nutrient broths with five different nutrient supplements were 299 and 260 nm in the corn broth, 256 nm in the soya bean broth, 222 nm in the sweet potato broth, 229 nm in the watermelon seed broth and 235 nm in the groundnut broth. Hence by comparing all the peaks in the nutrient broths that amended with corn had the highest absorbance peak compared to the others. The maximum production range of pyocyanin in the nutrient broth was at 229 and 260 nm in the corn broth and the minimum production was at 222 nm in the sweet potato broth.

Similarly, the absorbance peaks seen in the five different nutrient supplement broths were 299 and 260 nm in the corn, 256 nm in the soya bean, 222 nm in the sweet potato, 229 nm in the watermelon seed and 235 nm in the groundnut broths respectively (Supplementary Fig. 1).

**Figure 4:**
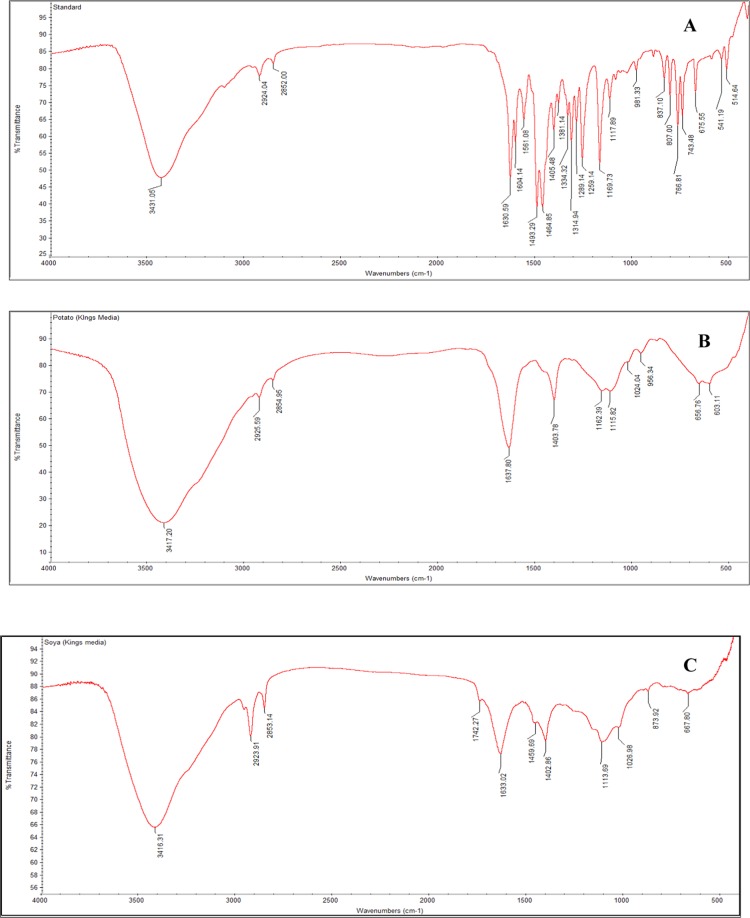
Functional groups spectral annotation by Fourier-Transform Infrared spectroscopy observed for standard pyocyanin (**A**), Sweet Potato (**B**) and Soya (**C**).

**Figure 5:**
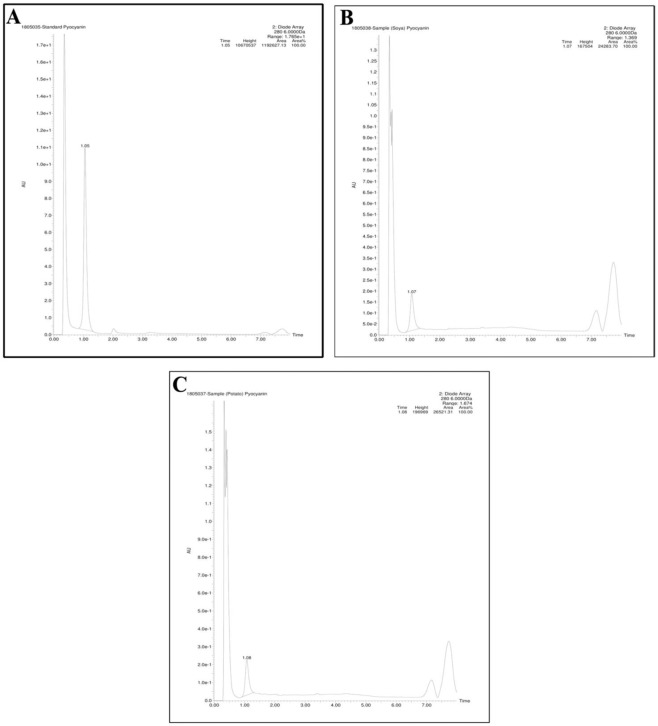
Characterization of pyocyanin by Liquid Chromatography Mass Spectra, Standard pyocyanin peak eluted at 1.05 mins (**A**), 1.07 mins for Soya sample (**B**) and and1.07 mins for Sweet potato (**C**).

**Figure 6:**
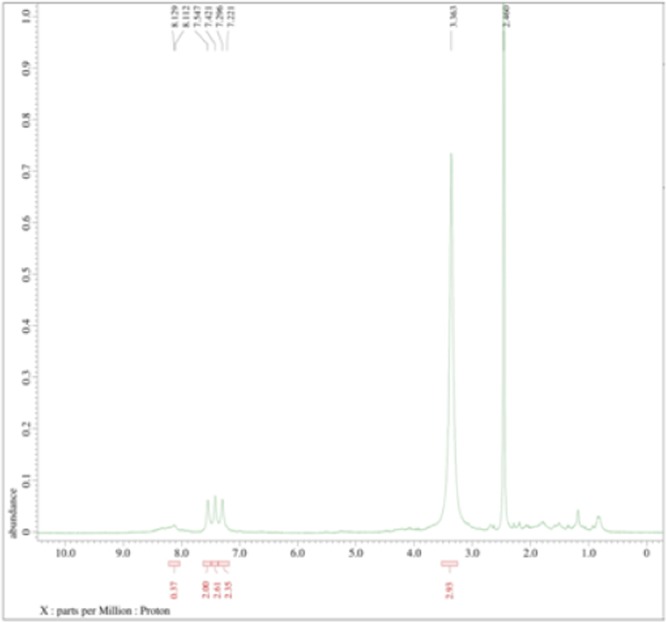
Structural elucidation of pyocyanin by Nuclear Magnetic Resonance Spectrum resonating at 3.363 ppm as a singlet.

The standard pyocyanin FTIR data revealed the presence of an Alkenyl C=C stretch at 1604, a C=O-H stretch Aldehyde at 1405 and a C-C-C stretch, with a medium stretch of 1169. Corresponding to the standards, the test samples exhibited similar molecular functional groups at 1604, 1405 and 1169 (Fig. 4). The standard pyocyanin peak was eluted at 1.05 mins with an m/z value of 211.05 and production of 197.04. Similarly, the extracted samples peaks were also eluted at 1.08 and 1.07 for sweet potato and soya bean respectively (Fig. 5). The 1 H NMR spectrum of pyocyanin was recorded with DMSO-d6 and N-CH_3_ protons resonated at 3.363 ppm as a singlet, whereas the remaining protons were resonated in the aromatic region from 7.221 to 7.547 ppm as multiplets (Fig. 6).

#### Antimicrobial properties of pyocyanin

**Figure 7:**
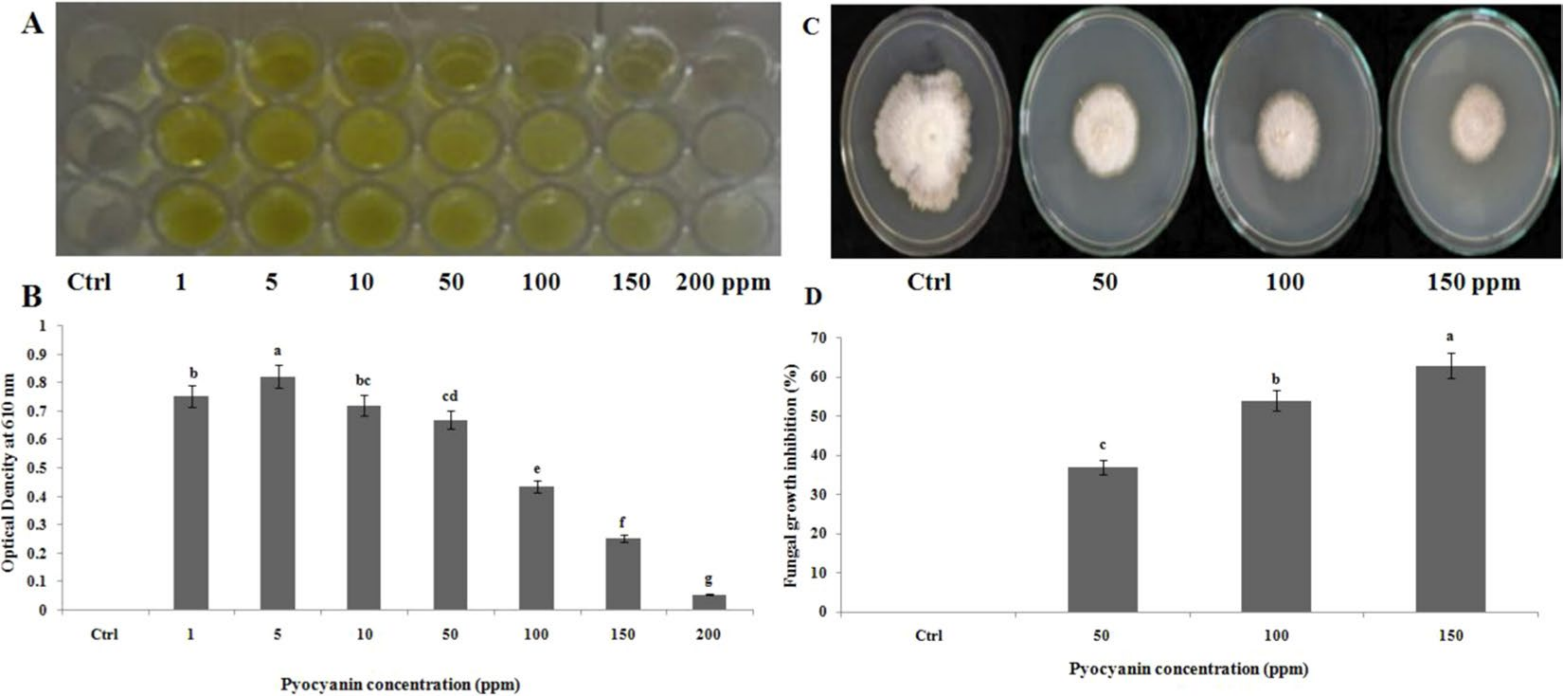
Agrochemical properties of extracted pyocyanin. (**A**) and (**C**) photographs exhibiting growth inhibiting action at 200 ppm against *Xanthomonas oryzae* (**A**) and at 150 ppm against *Magnaporthe grisea* (**C**), respectively. Images (**B**,**D**) showing the quantification of *X*. *oryzae* load measured at 610 nm and (**D**) The diameter of zone of inhibition in *M*. *oryzae* growth. Values are the mean of four independent replicates ± standard errors (n = 4). Significance of the data are measured according to Tukey’s HSD test.

In the antimicrobial susceptibility tests, pyocyanin showed microbial growth inhibiting activity against a tested fungus and a bacterium. Pyocyanin was introduced to the pathogens in different concentrations 1, 5, 10, 50, 100, 150 and 200 ppm. The bacterial pigment pyocyanin reduced the growth of the fungus *Magnaporthe grisea* and the bacterium *Xanthomonas oryzae* and it was noticed that at 200 ppm the growth was completely inhibited. The growth of the fungus was inhibited to the maximum extent at 150 ppm (Fig. 7).

#### Pyocyanin as a textile dye

**Figure 8:**
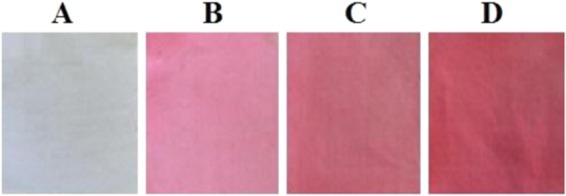
Textile dye application of extracted pyocyanin pigment on white cotton cloth. Cloth without the application of pyocyanin (**A**), cloth washed after the six hours of pyocyanin application (**B**), cloth washed with soap after 12 h of pyocyanin application (**C**), cloth washed with water after 12 h of treatment and cloth showing higher dye accumulation after 24 h of pyocyanin application (**D**).

The extracted pyocyanin showed the property of a textile dye and the results obtained using cotton cloth are shown in the Fig. 8. The cotton cloth treated with pyocyanin pigment extract showed the change in colour from white to pink. After dye application and 3–5 times washing with soap, the color change remained.

## Discussion

*Pseudomonas aeruginosa* is an opportunistic bacterium with commercial value and it is well known for its ability to secrete phenazine compounds^18^. It is responsible for broad spectrum of activities, such as dye decoloration^19^, food colorants^20^, antibacterial^20^, antifungal^21^, nematocidal^21^ and pesticide degradation^22^. Moreover, *P*. *aeruginosa* has also been documented for its efficacy in plant growth promotion and is a promisive biocontrol candidate^23^. This microorganism exhibits a versatile biological activity which can be utilized for solving many agricultural^20,21,23^ and environmental issues^24^.

In this study, seven bacterial strains were isolated from agricultural waste soil and screened for pyocyanin production. Among the seven bacteria, isolate KU-BIO2 was found to be promising in pyocyanin production, and based on the 16s rRNA analysis it was identified as *P*. *aeruginosa* (Fig. 1). The sequence readings of the isolate KU-BI02 showed high similarity with various other isolates of *P*. *aeruginosa* that had been previously reported for beneficial application such as bioremediation or oil degradation and biosurfactant production (Fig. 1). In this study, we also noticed a enhanced pyocyanin production by this isolate KU-BIO2 that was achieved by amendment of the nutrients in the growth medium, and maximum production was obtained in King’s A broth (Table 1) when compared to nutrient broth (Table 2). It was observed that the production of pyocyanian is nutrient dependent, the amendement of natural nutrients such as carbohydrate, fat, minerals and proteins in the form of soya bean in King’s A growth medium carrying peptone, magnesium chloride and glycerol as a major chemical nutrients influence the production of pyocyanin, these combined natural and chemical nutrients in King’s A medium play a vital role by increasing the bacterial density and would produce maximal amounts of pyocyanin pigment. While, nutrient broth is provided with low energy sources. Therefore, we reason that the pigment is contributed in association and or dependent with supplemented natural nutrients. Similarly, Devnath *et al*.^25^ observed the maximum pyocyanin pigmentation from the growth medium amended with peptone, magnesium chloride and glycerol, and increase pigment formation was observed in combination with magnesium chloride and inorganic ions. In the past, various researchers reported the usage of traditional low cost carbon sources for *Pseudomonas* spp. growth and higher pyocyanin production. For instance, molasses as a rich carbon act as a growth promoting substrate for *Pseudomonas* spp. and pyocyanin production^26^. Similar investigation was performed by E-Fouly *et al*.^27^ in which pyocyanin production from *P*.*aeuroginosa* was carried out using glycerol supplemented nutrient broth (GSNB), Mineral Medium and King’s A broth and it was concluded that the production of pyocyanin was enhanced by using raw materials as nutrient sources. These results support our conclusion that the production of pyocyanin is nutrient dependent and the microbes have an advantage in synthesis of important pigments by the use of a variety of raw nutrients under varying cultivation processes^27,28^. Hence, based on our results it can be considered as a low cost chemical and natural nutrients are essential for the microbial growth and metabolism underlying the glycolysis pathway, where microbes used food for the energy to run cellular processes, thereby bestow enhanced production of pyocyanin.

On the contrary, pyocyanin is synthesized commercially using chemical reagents such as phenazine methasulfate, nitrogen, chloroform, hexane, methanol, millipore water, hydrochloric acid Tris-HCL and sodium hydroxide^29^. Such chemical synthesis is expensive and time consuming. To overcome this issue, in the present investigation the pyocyanin was naturally produced from the *P*.*aeuroginosa* strain KU-BIO2 within three days. Likewise, E-Fouly *et al*.^27^ reported maximum pyocyanin production from *P*.*aeuroginosa* after the fourth day. Although synthesis of natural pigment is time consuming, pyocyanin has been successfully extracted using a microbial source within a short period using simple raw nutrients. Moreover, the media plays a crucial role in the microbial growth and enhanced production of pyocyanin, and a number of synthetic media are strongly suggested for pyocyanin production under low proliferation of *P*. *aeruginosa*^21^. Interestingly, the *P*. *aeruginosa* oxyR mutant is unable to grow in LB medium unless high quantities of cells are inoculated. Conversely, the growth in King’s A media is found to be optimum with induced pyocyanin and rhamnolipid production^30^. In the present study, evidence is provided for the improved growth and cell proliferation of *P*. *aeruginosa* in King’s A medium when compared to the Nutrient broth. Being a versatile agent, *P*. *aeruginosa*is able to utilize various types of carbon sources for the production of rhamnolipids^31^.

The absorbance peak of standard pyocyanin was eluted at 382 and 521 nm against 0.2 M HCL as a blank (Fig. 3A). Likewise, the pyocyanin extracted from King’s A media supplemented with corn, soya bean or sweet potato showed elution at 384 nm (Fig. 3B–D) and confirms pyocyanin presence in the media. Other pyocyanin characteristic peaks under UV-Vis spectrophotometer examination are in agreement with Ohfuji *et al*.^32^. Similarly, Kerr *et al*.^15^ reported the use of the UV-Visible spectrophotometer for detection of pyocyanin pigment successfully extracted from different growth media. The isolated pyocyanin was characterized by LCMS and the molecular ion of pyocyanin was found at 211 m/z, this data outcome being supported by E-Fouly *et al*.^27^. Furthermore, the 1 H NMR spectrum of pyocyanin was recorded by DMSO-d6 and N-CH_3_ protons which were resonated at 3.363 ppm as a singlet, and others were resonated in the aromatic region from 7.221–7.547 ppm (Fig. 6), and this result is in accordance with the earlier report^33^.

In the present study, pyocyanin was found to be effective against *Magnaporthe grisea* and *Xanthomonas oryzae* at 150 and 200 ppm, respectively (Fig. 7). Abdul-Hussein & Atia^34^ reported that the growth of *Escherichia coli* and of *Aspergillus fumigatus* were inhibited in the range between 10 to 100 μg ml^−1^, while the growth of yeast *Cryptococcus neoformans* was equally inhibited by pyocyanin. In another study, *E*. *coli*, *Bacillus sp*., and *Aspergillus niger* strains were found to be sensitive to the phenazin pigment pyocyanin at 0.005 to 50 µg as analyzed by the tube dilution and agar well diffusion methods^35^. The antagonistic activity of pyocyanin was studied at low concentration of 100 µL against *Macrophomina phaseolina* plant pathogenic fungus that is responsible for stem rot, seedling blight and root rot. These authors noticed that the pyocyanin was exhibited as a tremendous growth inhibiting agent against *M*. *phaseolina* and could suppress the associated diseases very effectively at low concentration, and the inhibitory action of 100 µl of purified pyocyanin against various phytopathogens and saprophytic molds was also reported^36^. The quorum sensing *P*. *aeruginosa* producing pyocyanin is correlated with siderospores activity that possesses antibacterial property^37^. In another independent research, it was observed that the phenazin pigment 50 µg produced by *P*. *aeruginosa* inhibit the growth of *Candida albicans* and *Aspergillus fumigatus* fungal growth^38^. The broad spectrum of antagonistic and biocontrol properties of pyocyanin isolated from *P*. *aeruginosa* is well documented^39^. It is also reported that pyocyanin isolated from oil degrading *P*. *aeruginosa* strain exhibit remarkable antimicrobial properties^40^.

Many of the microbial mediated compounds not only show antimicrobial activities but also possess ecofriendly coloring agents that can be used in food processing, textile and cosmetic industries with high demand^41–43^. In the past there have been well documented reports about the production of bio-pigments, namely flavins, quinines, carotenoids and melanins, from various microbial origins^42,44^. However, in the Indian scenario, very limited research results on the utilization of microoranisms for the production of pyocyanincolor pigments are available. In this context and in view of an eco-friendly industrial approach, synthetic dyes are becoming expensive and causing many harmful effects on the environment and hence nowdays research on natural and biological dyes is gaining much attention^45^. Microbial pigments are regularly characterized as industrial dyes and coloring agents in preparation of foods and paints^46,47^. Additionaly, some microbial strains are used as a textile colorants. For example *Flavobacterium* sp.^48^, *P*. *aeruginosa* and *Serratia marcescens*^49^ produce distinct colors. Likewise, in the current study the *P*. *aeruginosa* KU-BIO2 strain was found to produce pyocyanin pigment effects on white cotton cloth producing a pink color (Fig. 8) which has a significant role to play in textile industries. Though, pyocyanin is widely used in industrial and agricultural applications, there are adverse effects of pyocyanin on human health such as cardiovascular, respiratory and nervous systems^50^. In another study, pyocyanin induces the cytotoxicity in human embryonic lung epithelial cell line^51^ and mammalian cells^52^.

The findings from these studies clearly illustrate that the pyocyanin can act as a potential agrochemical properties which can reduce the use of toxic fungicides. Further, evidence is provided about the use of pyocyanin pigment in textile industries such as it being a natural colorant for materials in the manufacture of fabrics and carpets, as well as coloring paper, and the possibility exists with using in textile and dye making industries.

## Methods

### Chemicals and soil samples

Standard pyocyanin with a purity of 98% was procured from the Cayman Chemical Company, USA. The chloroform and hydrochloric acid used were of high analytical grade, and for the isolation of *Pseudomonas aeruginosa*, Citrimide broth (CB), Nutrient agar (NA) and King’s A medium (Himedia, Mumbai, India) were utilized. The soil samples were collected from where agricultural waste was dumped in Dharwad. Karnataka, India.

### Isolation of *Pseudomonas* species

One gram of finely sieved soil sample was serially diluted up to 10^−7^ using sterile saline solution, and 100 μl of 10^−5^ and 10^−6^ dilutions were aseptically inoculated to tubes containing 10 ml CB and incubated at 35 °C for 24 hrs. After the incubation, an aliquot of 100 μl was spread plated on the NA and incubated at 35 °C for 24 hrs. Further, the fully grown colonies were purified by using the quadrant streak method and stored at 4 °C until needed for further use.

### Characterization of *Pseudomonas* species

All the isolates were screened initially by their colony morphology and gram nature, and biochemically by testing the positive interaction with oxidase and catalase, gelatin liquefaction and citrate utilization tests. Additionally, the ability of the isolates to grow at high temperatures (40 to 45 °C) was also investigated. Further, *Pseudomonas* spp. was confirmed by the pigment coloration (fluorescent glow) on the NA. The cells of *Pseudomonas* spp. grown in CB were fixed on a clean glass slide and were scanned at different resolution ranges using Flex–Bio Atomic Force Microscope (AFM) (Nanosurf) with C3000 controller for cell morphology analysis. Later, the isolate was subjected to molecular characterization with 16S rRNA universal primer in a thermal cycler^2^.

### Nutrient sources for the enhanced production of the pyocyanin pigment

Five nutrient sources (NS) were selected for the enhanced production of pyocyanin. NSs like sweet corn, sweet potato, watermelon seeds, peanuts and soya seeds were chosen. Sweet corn was washed with distilled water, finely ground and stored in the refrigerator until used, the sweet potato was boiled in water and the boiled water filtered and used. Watermelon seeds, soya seeds and peanuts were washed, dried and finely ground using mortar and pestle and stored in a polythene bag until further use.

### Enhanced production of pyacyanin pigment

The enhanced production of pyocyanin was done using Nutrient Broth (NB) and King’s A medium. A 100 µl of liquid culture consisting of cell density 3 × 10^6^ cfu ml^−1^ was inoculated to 100 ml NB and King’s A medium supplemented with one percent of NS and incubated at 35 °C in a shaker incubator (160 rpm) up to log phase. Bacteria grown without NS were treated as control and the growth of the strain was monitored during the study.

### Extraction of pyocyanin by *Pseudomonas* species

At the log phase of bacterial growth, the broth culture was withdrawn from each of the flasks and subjected to centrifugation at 12,000 × g for 20 min at 4 °C. The supernatant was then collected and thoroughly mixed with 4.5 ml of chloroform and vortexed for 20 secs for colour change, and the biomass which was settled at the bottom was dried at 75 °C and the obtained dried biomass was considered weight per volume. Then, (chloroform sinks to the bottom of the tube, make sure that the supernatant moves with chloroform when vortex, the color of chloroform changes from green to blue). Samples were centrifuged at 10,000 × g for 10 mins at 4 °C and then three ml of the blue layered solution settled at the bottom of the tube were transferred to another new set of tubes and 1.5 ml of 0.2 M HCl was introduced to each tube, properly mixed, and further subjected to centrifugation for five mins at 10, 000 × g, the resultant suspension containing pigment was stored in 4 °C until further use. The supernatant obtained was utilized to measure the density of extracted crude pyocyanin at different wavelengths in a spectrophotometer (Hitachi U2900). The intensity of pyocyanin concentration (µg ml^−1^) was calculated by multiplying the optical density (OD) value obtained at 520 nm.

### Purification of pyocyanin pigment

The crude pyocyanin pigment was purified by the method described in E-Fouly *et al*.^27^. Briefly, the crude pigment was suspended in chloroform and absorbed on silica gel. Further, the absorbed pigment samples were loaded onto the column which was equilibrated by methanol and chloroform and later the elution of pure pigment was done using a combination methanol and chloroform.

### Chemical analysis

#### Confirmatory test for the production of pyocyanin by UV spectroscopy

The obtained pigment was re-dissolved in five ml of chloroform and utilized to measure the density of the extracted pyocyanin at different wavelengths (300–700 nm) using a spectrophotometer (Hitachi U2900). The intensity of pyocyanin concentration (µg ml^−1^) was calculated by multiplying the optical density (OD) value obtained at 520 nm.

#### Identification of functional groups by Fourier-Transform Infrared spectroscopy (FTIR)

The extracted pyocyanin samples from different sources was encapsulated in 200 mg of KBr (Sigma-Aldrich) in order to prepare translucent sample disks homogenized with KBr. The spectra were measured using a Nicolet FT-IR 6700 (Thermo Fisher Scientific) in the IR region, a single beam splitter, and a DTGS-KBr detector at spectrometer range 4000 to 400 cm^−1^.

#### Characterization of pyocyanin by Liquid Chromatography–Mass Spectrometry (LCMS)

An aliquot of standard and extracted pyocyanin was used for the LCMS analysis. Ten µl of analyte was subjected to LC (Aquity, Waters) equipped with BEH C18 of 1.7 µm of a 1.0 × 50 mm column and followed by mass spectrometry, the mass range being ionized by the Synapt G2 Waters. An analyte prepared with 0.1% formic acid in water in combination with acetonitrile was subjected to LC at controlled room temperature and column temperature of 50 °C in gradient mode for 8 mins reverse phase.

#### Elucidation of pyocyanin structure by NMR

The lyophilized sample of pyocyanin was checked for its solubility and it was clearly soluble in DMSO solvent. Ten mg of the sample was dissolved in the NMR tube containing DMSO solvent, the DMSO solvent was filled till it reached about 4–5 cm of the tube. The tube was gently shaken for all the material to dissolve. Then the NMR tube was inserted into a spinner and was taken out from the spinner after applying a magnetic field and was placed in the NMR spectrometer.

#### Antimicrobial assay of pyocyanin

The antifungal assay of pyocyanin was examined by the method of Jogaiath *et al*.^4^ and Joshi *et al*.^53^. Briefly, rice blast caused by the *Magnaporthe grisea* and bacterial blight of rice, *Xanthomonas oryzae*, were tested against the pyocyanin on Potato Dextrose Agar (PDA) and Nutrient Broth medium (NB) respectively. PDA petri dishes supplemented with pyocyanin at 2.560 µg ml^−1^ concentration were inoculated with *Magnaporthe grisea* and incubated for five days at 25 ± 2 °C. PDA petri plates and 96-well microtiter plates without pyocyanin served as the control. The minimal inhibitory concentration (MIC) value of *Xanthomonas oryzae* was analysed for pyocyanin and this was determined by a micro dilution method with the initial cell load of 5 × 10^5^ CFUml^−1^ and measured using spectrophotometer at 610 nm.

#### Application of pyocyanin as a textile colorant

The scope for probable application of the bacterial pigment was evaluated on textile white cotton material commercially available in the market. The bacterial pigment was applied to the cloth material which was then washed with soap solution, rinsed with tap water to remove the unfixed dye substances present at the surface level, dried at room temperature and ironed^17^.

### Ethical approval

This article does not contain any studies related to human participants or animals.

## Supplementary information

Supplementary information.
