# Supplementary material for: Isolation and characterization of nutrient dependent pyocyanin from Pseudomonas aeruginosa and its dye and agrochemical properties
Source: Sci Rep. 2020 Jan 31;10:1542. doi: 10.1038/s41598-020-58335-6 (PMC6994680; doi:10.1038/s41598-020-58335-6)
Supplement: Supplementary file 1 — Supplementary information. [file 41598_2020_58335_MOESM1_ESM.docx]

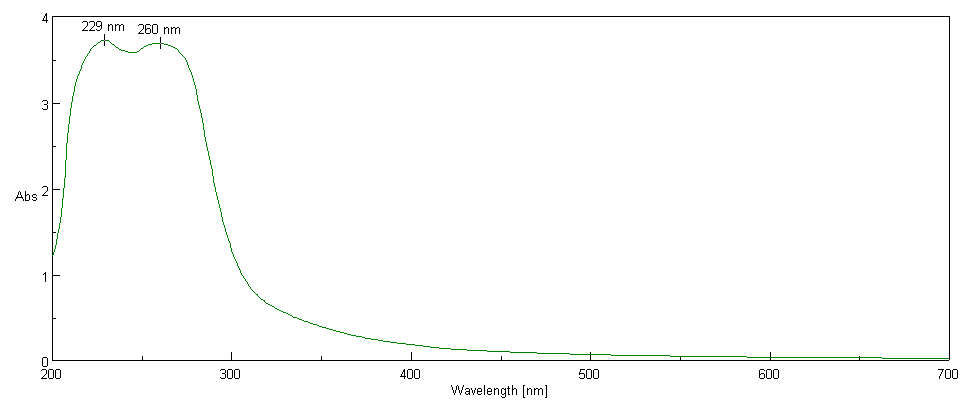

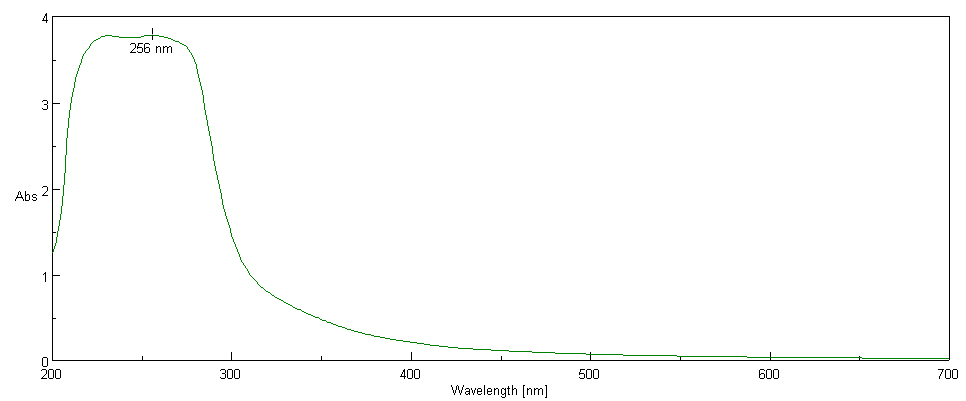

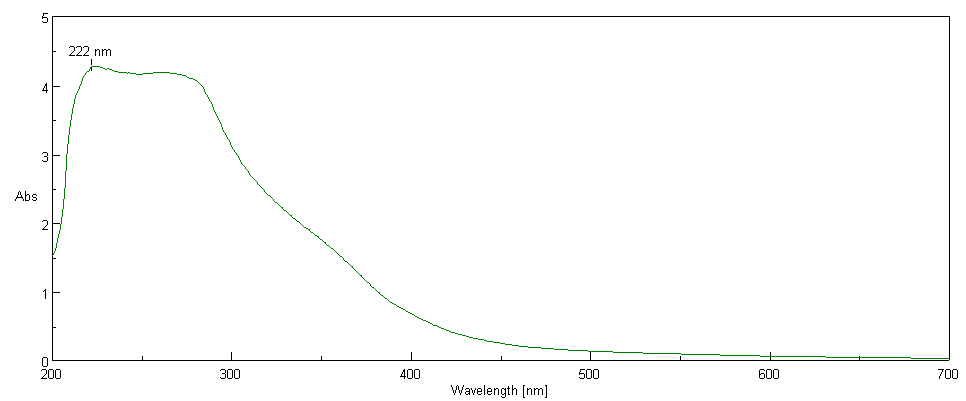

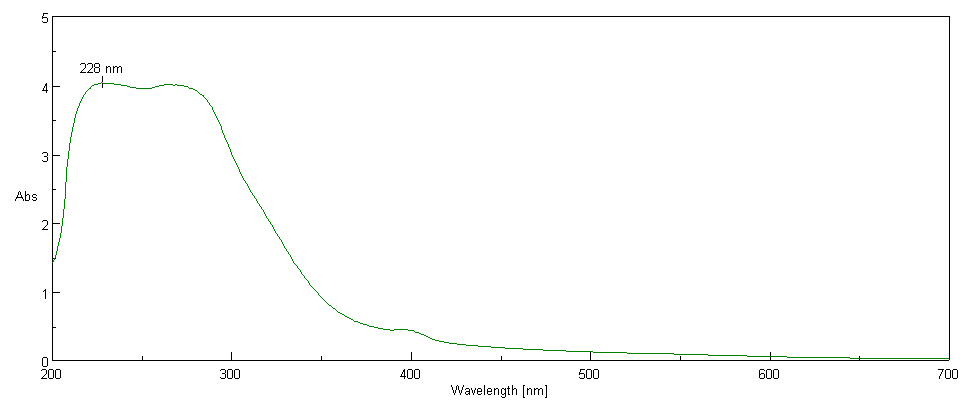


**A**

**B**

**C**

**D**

**Supplementary Fig. 1.** Absorbance peaks of pyocyanin extracted from the nutrient broth amended with corn (A), soya (B), sweet potato (C) and water melon (D) as an energy sources for the production of pyocyanin.
